# Cross-sectional and longitudinal characterization of SCD patients recruited from the community versus from a memory clinic: subjective cognitive decline, psychoaffective factors, cognitive performances, and atrophy progression over time

**DOI:** 10.1186/s13195-019-0514-z

**Published:** 2019-07-08

**Authors:** Elizabeth Kuhn, Inès Moulinet, Audrey Perrotin, Renaud La Joie, Brigitte Landeau, Clémence Tomadesso, Alexandre Bejanin, Siya Sherif, Vincent De La Sayette, Béatrice Desgranges, Denis Vivien, Géraldine Poisnel, Gaëlle Chételat

**Affiliations:** 1 Inserm, Inserm UMR-S U1237, GIP Cyceron, Université de Caen-Normandie, Boulevard H. Becquerel, 14000 Caen, France; 2 Memory and Aging Center, Department of Neurology, University of California, San Francisco, San Francisco, CA USA; 3 Normandie Univ, UNICAEN, PSL Recherche Universités, EPHE, INSERM, U1077, CHU de Caen, Neuropsychologie et Imagerie de la Mémoire Humaine, GIP Cyceron, 14000 Caen, France; 4 CHU de Caen, Service de Neurologie, Caen, France; 5 Department of Clinical Research, Caen Normandy Hospital (CHU) de Caen, 14000 Caen, France

**Keywords:** Neuroimaging, Biomarkers, Pathological ageing, Preclinical Alzheimer’s disease, Psychoaffective factors, Subjective cognitive decline

## Abstract

**Background:**

Subjective cognitive decline (SCD) defines a heterogeneous population, part of which having Alzheimer’s disease (AD). We aimed at characterizing SCD populations according to whether or not they referred to a memory clinic, by assessing the factors associated with increased AD risk.

**Methods:**

Seventy-eight cognitively unimpaired older adults from the IMAP+ study (Caen) were included, amongst which 28 healthy controls (HC) and 50 SCD recruited from the community (SCD-community; *n* = 23) or from a memory clinic (SCD-clinic; *n* = 27). Participants underwent cognitive, psychoaffective, structural MRI, FDG-PET, and amyloid-PET assessments. They were followed up over a mean period of 2.4 ± 0.8 years. The groups were compared in terms of baseline and follow-up levels of SCD (self- and informant-reported), cognition, subclinical anxiety and depression, and atrophy progression over time. We also investigated SCD substrates within each SCD group through the correlations between self-reported SCD and other psychometric and brain measures.

**Results:**

Compared to HC, both SCD groups showed similar cognitive performances but higher informant-reported SCD and anxiety. Compared to SCD-community, SCD-clinic showed higher informant-reported SCD, depression score, and atrophy progression over time but similar brain amyloid load. A significant increase over time was found for depression in the SCD-community and for self-reported praxis-domestic activities SCD factor in the SCD-clinic. Higher self-reported SCD correlated with (i) lower grey matter volume and higher anxiety in SCD-community, (ii) greater informant-reported SCD in SCD-clinic, and (iii) lower glucose metabolism in both SCD groups.

**Conclusions:**

Higher subclinical depression and informant-reported SCD specifically characterize the SCD group that refers to a memory clinic. The same group appears as a frailer population than SCD-community as they show greater atrophy progression over time. Yet, both the SCD groups were quite similar otherwise including for brain amyloid load and the SCD-community showed increased depression score over time. Altogether, our findings highlight the relevance of assessing psychoaffective factors and informant-reported SCD in SCD populations and point to both differences and similarities in SCD populations referring or not to a memory clinic.

**Electronic supplementary material:**

The online version of this article (10.1186/s13195-019-0514-z) contains supplementary material, which is available to authorized users.

## Background

Subjective cognitive decline (SCD) refers to individuals’ perceived decline in memory and/or other cognitive abilities relative to their previous level of performance, in the absence of objective neuropsychological deficits [1]. Although these individuals have been described for decades [2], they have received increasing attention over the past few years due to growing interest in characterizing the preclinical stages of Alzheimer’s disease (AD) [3, 4]. Recent cross-sectional studies have shown that SCD is associated with neuroimaging biomarkers suggestive of AD such as hippocampal/parahippocampal atrophy [5–17] and/or temporoparietal hypometabolism [5, 18, 19], and cortical amyloid β (Aβ) deposition [18, 20–24] (see [25] for review). Longitudinal investigations have repeatedly shown that SCD is also associated with an increased risk of subsequent cognitive decline [26, 27] or conversion to mild cognitive impairment (MCI) or AD dementia [28–34]. There is thus converging evidence that SCD is associated with an increased risk of AD dementia and might represent, at least for some cases, the first clinically observable sign of Alzheimer’s clinical syndrome [35].

However, the links between SCD and AD biomarkers have not been reported in all studies (see [25] for review), which might reflect the fact that SCD is multi-determined. Thus, SCD may be due to AD but also non-AD aetiologies (see [36] for review, [37]) including normal ageing [38], poor general health [38, 39], medication [1], sleep disorders [40–43], or psychoaffective factors such as anxiety and depression [39–41, 44]. The current challenge, as highlighted in an international collaborative working group on SCD called the SCD-Initiative (SCD-I; [1, 45]), is thus to identify the specific characteristics of SCD that are associated with an increased likelihood of AD aetiology [1, 45].

An important source of heterogeneity in the definition and aetiology of SCD patients is their type of recruitment [25] (see [46–48] for review). Indeed, typical research settings include population-based studies [6, 24], volunteer samples [18, 20, 21, 23], and/or medical help-seeking samples [5, 7–12, 22]. In a previous study [25], we showed that SCD patients who refer to a memory clinic, referred to as SCD-clinic, had significant atrophy in AD-sensitive regions compared to SCD individuals recruited from a self-reported SCD questionnaire in volunteers from the community, referred to as SCD-community in what follows. This previous study suggested that SCD-clinic as a group is further along the Alzheimer’s clinical syndrome trajectory than SCD-community.

In this study, our aim was to provide further evidence towards this statement with a more complete characterization of both SCD-clinic and SCD-community populations of the substrates of their SCD and of their evolution.

For this purpose, we first highlighted the similarities and differences between the two SCD populations in terms of cognitive performances, psychoaffective measures, informant-reported SCD, and the type of self-reported SCD. Informant-reported SCD is recognized as a feature that influences the likelihood of preclinical AD/Alzheimer’s syndrome—confirmation of cognitive decline by an informant being associated with increased risk for preclinical AD/Alzheimer’s syndrome [1, 45, 49, 50]. Moreover, subclinical symptoms of anxiety or depression might be related with SCD and may constitute risk factors for subsequent cognitive decline and/or be early manifestations of preclinical Alzheimer’s syndrome [51–55]. In addition, a previous study showed that SCD of different cognitive domains were differentially associated with preclinical Alzheimer’s syndrome [56].

Second, we investigated the substrates of self-reported SCD within each SCD group by assessing the correlations with informant-reported SCD, psychoaffective and cognitive measures, and neuroimaging biomarkers (grey matter atrophy, cerebral glucose hypometabolism, and amyloid deposition).

Finally, we studied the evolution of the groups over a mean follow-up period of 2.4 years in terms of SCD, psychoaffective and cognitive measures, and atrophy progression over time. We also assessed how baseline variables predicted subsequent cognitive decline.

## Methods

### Subjects

A total of 78 cognitively unimpaired individuals were included from the *Imagerie Multimodale de la maladie d’Alzheimer à un stade Précoce* (IMAP+) study (Caen, France). The inclusion and exclusion criteria are detailed in previous publications. Briefly, participants were all aged over 50 years; had at least 7 years of education; had no history of alcoholism, drug abuse, head trauma, or psychiatric disorder; and performed in the normal range on a standardized neuropsychological examination [8, 25, 57].

**Fig. 1:**
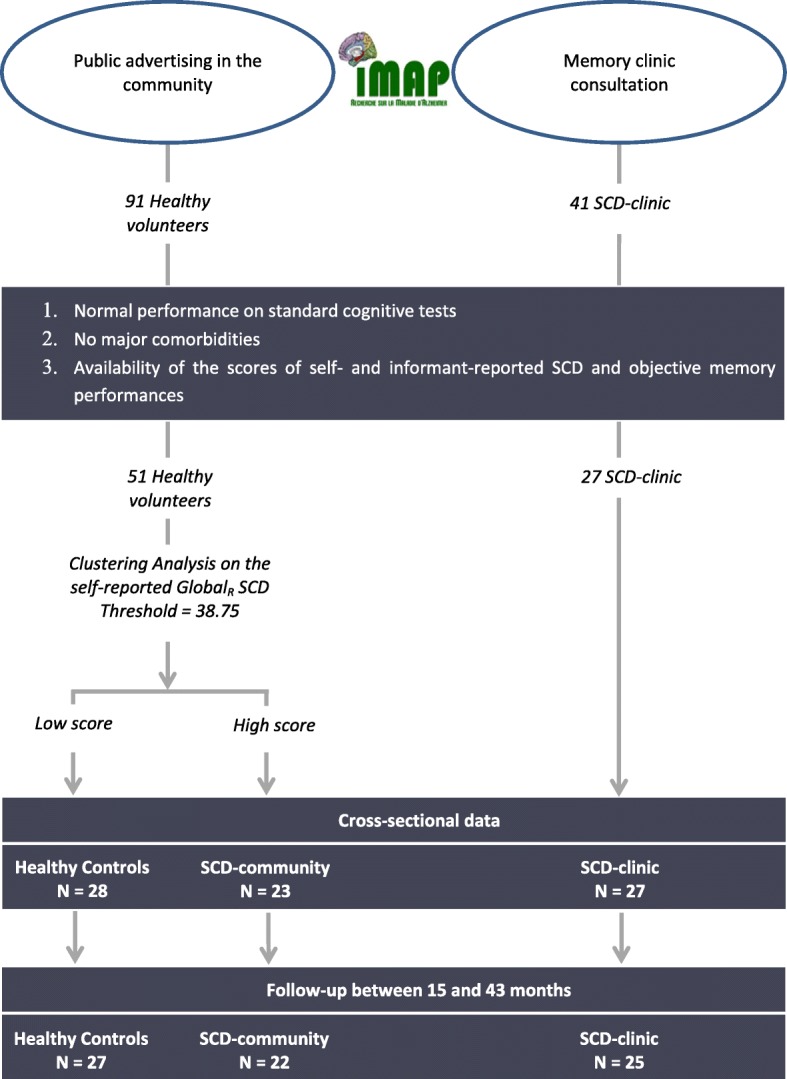
A flow chart of participant selection and categorization. The graphic shows the steps to select the participants finally included in the three groups of interest of the present study—the healthy controls and the cognitively unimpaired older adults with subjective cognitive decline (SCD) who referred (SCD-clinic) or not (SCD-community) to a memory clinic. All participants were selected from the *Imagerie Multimodale de la maladie d’Alzheimer à un stade Précoce *(IMAP+) study. *N*, sample size

Participants were recruited from two main sources, memory clinic or public advertising (see Fig. 1). The first group of SCD patients was recruited from a local memory clinic consultation (SCD-clinic) that they attended because of memory concerns. The clinical diagnosis was obtained by a multidisciplinary consensus under the supervision of a senior neurologist. The subjective cognitive decline was self-reported to the clinician during the interview and with a 10-item SCD questionnaire, the Cognitive Complaint Questionnaire [58]. Before inclusion, the clinician checked that the SCD was not caused by medication, psychoaffective conditions (including a major depressive disorder or generalized anxiety disorder), or other medical conditions. Amongst the 41 patients who met these criteria, only those for which the main variables of interest (self- and informant-reported SCD questionnaires and objective episodic memory score) were available were included in the present study, resulting in a group of 27 SCD-clinic.

Fifty-one participants were recruited from the community through public advertising, as they volunteered to participate in the IMAP+ study. This group was subdivided into two groups based on their score of self-reported SCD (see below and Fig. 1) resulting in 28 participants with a low score considered as the healthy control (HC) and 23 participants with a high score labelled as the SCD-community. As for the SCD-clinic, only the participants for whom the three main measures of interest were available were included in the present study.

The IMAP+ study was approved by the local ethics committee. After the complete description of the study to the participants, written informed consent was obtained from all participants.

### Neuropsychological assessment

Neuropsychological assessment was performed on the same site for all participants at baseline and at follow-up. The mean duration between serial neuropsychological assessments was 2.4 (± 0.8) years.

### Self- and informant-reported cognitive difficulties

SCD was assessed with the Cognitive Difficulties Scale (CDS) [59], a 39-item self-rated questionnaire that requires participants to rate how often they experience particular cognitive difficulties in everyday life on a 5-point scale (from “never” = 0 to “very often” = 4). This questionnaire was performed by the participant about himself, resulting in a self-reported measure of SCD, and by the participant’s informant about the participant, resulting in an informant-reported measure of SCD. Higher scores indicate greater SCD.

In the present study, we used the reduced SCD score of the CDS [59], corresponding to the sum of 34 items, as a measure of self- or informant-reported cognitive difficulties. Five items were removed from the initial questionnaire which correspond to gendered items (e.g. related to cooking or sewing), as they depend on age-specific cultural norms [59]. This score will be referred to as ‘global_R_ SCD’ in the following article. The self-reported global_R_ SCD score was used to separate the HC from the SCD-community. More specifically, a hierarchical clustering analysis (2 clusters, 50 iterations) was performed on this score within the cognitively unimpaired volunteers recruited from the community, resulting in 28 participants with a low score (HC) and 23 participants with a high score (SCD-community).

In a previous study by our team, we conducted a factorial analysis on the CDS scale and highlighted three different factors reflecting different types of SCD [56]: (i) the first factor (F1) was composed of 11 items related to attention and language; (ii) the second factor (F2) included 12 items related to memory and orientation; and (iii) the third factor (F3) included 7 items related to praxis and domestic activities [56]. These three factors were computed for each participant using each item weighted according to the results of the factorial analysis from La Joie et al. [56] (for self- and informant-reported SCD) and compared between the groups.

### Psychoaffective measures

Depressive symptomatology and trait anxiety were assessed using the Montgomery-Asberg Depression Rating Scale (MADRS) [60] and Spielberger State-Trait Anxiety Inventory (STAI-B), respectively [61]. Higher scores indicated a higher level of depression or anxiety with all scores yet keeping within the subclinical levels as participants were screened for the lack of clinically significant anxiety or depression disorders.

### Cognitive measures

Global cognition was assessed using the Mini-Mental State Examination (MMSE) [62] and the global score of the Mattis Dementia Rating Scale (DRS) [63]. Memory was assessed using the Encoding, Storage and Recuperation (ESR) word list delayed recognition subscores [64].

### Cross-sectional data: transformation to *w*-scores

For cross-sectional analyses, all continuous raw scores were transformed into *w*-scores, which are age and education-adjusted *z*-scores relative to the control group [65], except for the psychoaffective measures.

### Longitudinal data: computation of slope of changes

For longitudinal analyses, a slope of decline was calculated for each measure of each subject with a simple linear regression equation ‘*y* = *ax* + *b*’ (where *y* is the score of interest; *x* is the number of months from the initial evaluation; *a* is the slope of the line; *b* is the intercept) [66].

### Neuroimaging assessment

#### Neuroimaging data acquisition

All participants were scanned on the same magnetic resonance imaging (MRI) and positron emission tomography (PET) cameras at the Cyceron Center (Caen, France): a Philips Achieva 3.0 T scanner and a Discovery RX VCT 64 PET-CT device (General Electric Healthcare), respectively.

High-resolution T1-weighted anatomical volumes were acquired using a 3D fast-field echo sequence (3D-T1-FFE sagittal; repetition time = 20 ms; echo time = 4.6 ms; flip angle = 10°; 180 slices with no gap; slice thickness = 1 mm; field of view = 256 × 256 mm^2^; in-plane resolution = 1 × 1 mm^2^). In the present study, we used the baseline and the follow-up MRI scans; the mean duration between serial MRI was 2.4 years (± 0.8 years). Follow-up MRI scan was missing in 1 HC, 1 SCD-community, and 3 SCD-clinic participants.

Both ^18^F-fluorodeoxyglucose (FDG) and florbetapir-PET scans were acquired with a resolution of 3.76 × 3.76 × 4.9 mm^3^ (field of view = 157 mm). Forty-seven planes were obtained with a voxel size of 1.95 × 1.95 × 3.2 mm^3^. A transmission scan was performed for attenuation correction before the PET acquisition. For ^18^F-FDG-PET, the participants were fasted for at least 6 h before scanning. After a 30-min resting period in a quiet and dark environment, 180 MBq of ^18^F-FDG was intravenously injected as a bolus. A 10-min PET acquisition scan began 50 min after the injection. For florbetapir-PET, each participant underwent a 20-min PET scan, beginning 50 min after the intravenous injections of ~ 4 MBq/kg of florbetapir. Two HC and 3 SCD-clinic participants only underwent a 10-min acquisition starting 50 min after the injection. In the present study, we used the baseline PET scans of the participants, which was missing for 1 HC for 18F-FDG-PET and for 3 HC and 1 SCD-community participants for florbetapir-PET (see Additional file 1).

#### Neuroimaging pre-processing

##### Cross-sectional data

Neuroimaging pre-processing was performed using the Statistical Parametric Mapping version 12 (SPM12) software (Wellcome Trust Centre for Neuroimaging, London, UK).

T1-weighted MRI were segmented using multimodal segmentation (T1-weighted MRI, T2-weighted MRI, and Flair) and spatially normalized to the Montreal Neurological Institute (MNI) space. Then, the normalized grey matter segments were modulated to correct for non-linear warping effects, and the resultant images were smoothed using an 8-mm full-width half-maximum (FWHM) Gaussian kernel [57, 67, 68].

PET data were corrected for partial volume effects using the Muller-Gartner method, coregistered onto their corresponding MRI, and normalized using the deformation parameters defined from the MRI procedure. Resultant images were quantitatively normalized using the cerebellar grey matter as the reference region. PET images were then smoothed using a 10-mm FWHM Gaussian kernel [57, 67–69].

All resultant MRI and PET images were finally masked to exclude non-grey matter voxels as well as the cerebellum from the analyses.

The global neocortical standardized uptake value ratio (SUVr) value was also obtained in each individual from the Florbetapir-PET SUVr images using a neocortex mask (including all regions but the cerebellum, hippocampus, amygdala, and subcortical grey nuclei). The SUVr was used to classify subjects as florbetapir positive or negative, using a threshold derived from an independent group of 41 young individuals from the IMAP project (16 females; age = 28.40 ± 6.06 years) [25, 70]. The positivity threshold was defined by the mean + 2 SD of 41 healthy young controls aged 21 to 39 years old (supposedly devoid of amyloid deposition), corresponding to a Florbetapir SUVr of 0.98. Individuals with values above this threshold were considered as amyloid-positive and those below this threshold as amyloid-negative.

##### Longitudinal changes

For each participant, a brain map of atrophy progression over time, reflecting the progression of atrophy over the follow-up period, was computed using the Jacobian determinants from the pairwise longitudinal registration of the baseline and follow-up MRI scans. The method is detailed in [71] and summarized in Additional file 2.

#### Statistical analysis

##### Cross-sectional data

To assess whether the control and both SCD groups differ in terms of demographic and clinical variables or self-reported global_R_ SCD, variance analyses (ANOVAs) with one three-level (group) factor were performed.

To highlight the differences between the three groups regarding the factors of interest potentially related to SCD or memory clinic consultation (i.e. psychoaffective measures, informant-reported SCD, types of SCD), we performed analyses of covariance (ANCOVAs) with one three-level (group) factor correcting for age and education, for all continuous variables—except for *w*-scores where ANOVAs were performed without covariates as age and education were already partialled out. Group differences for categorical variables were assessed using chi-square tests.

The cognitive substrates of self-reported SCD were assessed in each group using correlations, correcting for age and education for all variables but *w*-scores. Correlations were performed between self-reported global_R_ SCD score and self-reported SCD factors, informant-reported global_R_ SCD, cognitive performances (global cognition and memory *w*-scores), and psychoaffective measures (anxiety and depression). All statistical analyses of behavioural data were performed using the STATISTICA software (v13.0, StatSoft Inc., Tulsa, OK).

The cerebral substrates were assessed in each group using regression analyses correcting for age and education between the self-reported global_R_ SCD score and cross-sectional MRI and PET (FDG and Florbetapir) data. All statistical analyses of neuroimaging data were performed using the full factorial design in SPM12.

##### Longitudinal data

To determine whether SCD, cognitive, and psychoaffective measures significantly changed over time, the individual slopes of regression line were compared to zero for each SCD group separately, using one-sample *t* tests. Then, to assess whether the changes in these measures significantly differed between the groups, the slopes of regression line were compared between the groups using ANCOVAs with one two-level (group) factor, correcting for age and education, for each SCD, cognitive, and psychoaffective measures. Thirdly, to assess whether the atrophy progression over time significantly differed between the groups, an ANCOVA with one three-level (group) factor with age and education as covariates was performed in SPM12. Finally, to improve our understanding of the predictors of cognitive decline, regression analyses were performed within each SCD group between baseline SCD scores or psychoaffective measures and the slope of cognitive decline, correcting for age and education, using general linear models and the STATISTICA software.

Neuroimaging results are examined at *p*_uncorrected_ < 0.005 and cluster extent *k* > 250 mm^3^ also indicating results surviving the *p* < 0.001 and *k* > 50 mm^3^ threshold. This allows to take into account the clusters that were less significant but larger versus more significant but smaller. For behavioural results, when the main effect of the group was significant (*p* < 0.05), post-hoc analyses were performed using the Newman-Keuls method.

## Results

### Group characteristics

**Fig. 2:**
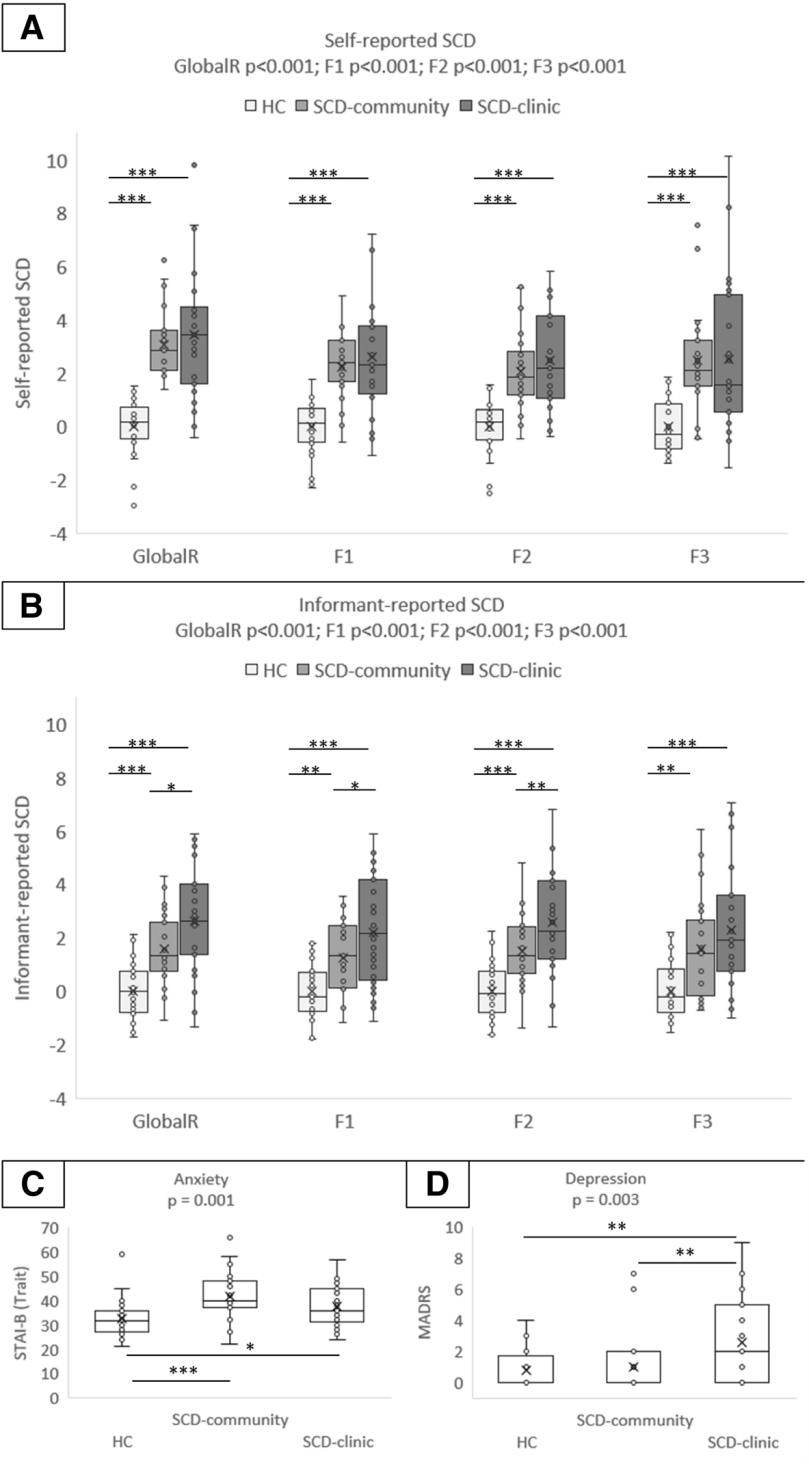
Group comparisons on subjective cognitive decline (SCD) and psychoaffective measures. The graphs indicate mean values and 95% confidence intervals. **a** Group comparisons on the global_R_ and the three self-reported SCD factors. **b** Group comparisons on the global_R_ and the three informant-reported SCD factors. **c** Group comparison on subclinical anxiety. **d** Group comparison on subclinical depression. **a**, **b** *p* corresponds to one-way analysis of variance (ANOVA); post-hoc analyses were performed with the Newman-Keuls difference test. Higher scores indicate greater SCD. **c**, **d** *p* corresponds to one-way analysis of covariance (ANCOVA) correcting for age and education; post-hoc analyses were performed with the Newman-Keuls difference test. Higher scores indicate higher subclinical anxiety or depression. *p < 0.05, **p < 0.01, ***p < 0.001 between the groups. F1, factor 1 attention-language SCD; F2, factor 2 memory-orientation SCD; F3, factor 3 praxis-domestic activities SCD; MADRS, Montgomery-Asberg Depression Rating Scale; STAI-B, Spielberger State-Anxiety Inventory Trait

There was no between-group difference in any demographic or cognitive variables; only a trend was found for SCD-clinic to be younger than HC and SCD-community to have a higher amyloid proportion of amyloid-positive individuals (Table 1). The self-reported global_R_ SCD score was higher in SCD-clinic compared to HC but equivalent in both SCD groups (Fig. 2a).

**Table 1:** Demographic features of the study populations

	HC	SCD-community	SCD-clinic	ANOVAs, *p* value
*N*	28	23	27	
Female % (*N*)	46 (13)	61 (14)	41 (11)	(*χ*^2^) NS^¤^
Age	72.25 ± 6.33	71.70 ± 6.60	68.30 ± 7.99	0.09
Level of education	11.50 ± 3.64	12.65 ± 4.13	12.85 ± 3.60	0.37
MMSE	28.68 ± 1.09	28.70 ± 1.18	28.70 ± 1.27	0.99
DRS	0.05 ± 1.02	0.08 ± 0.58	−0.12 ± 0.90	0.68
ESR	− 0 ± 0.71	0.01 ± 0.72	− 0.49 ± 1.70	0.18
APOE ε4 (carrier) % (*N*)	18 (5)	26 (6)	15 (4)	(*χ*^2^) NS^¤^
Amyloid status (pos) % (*N*)	22 (6)	45 (10)	33 (9)	(*χ*^2^) NS^¤^
SUVr	0.97 ± 0.17	1.03 ± 0.18	1.01 ± 0.19	0.46

Values indicate mean ± SD or percentage. When the analyses of variance (ANOVAs) reached significance, Newman-Keuls tests were used. ^¤^For gender, HC – SCD-community *p* = 0.29, HC – SCD-clinic *p* = 0.71, SCD-community – SCD-clinic *p* = 0.16; for APOE4 carrier: HC – SCD-community *p* = 0.49, HC – SCD-clinic *p* = 0.76, SCD-community – SCD-clinic *p* = 0.33; for amyloid status: HC – SCD-community 0.09, HC – SCD-clinic *p* = 0.37, SCD-community – SCD-clinic *p* = 0.39

*Abbreviations*: *APOE* ε4 apolipoprotein E allele 4, *DRS w*-score of Mattis Dementia Rating Scale, *ESR w*-score of Encoding, Storage and Recuperation, *HC* healthy control, *MMSE* Mini-Mental State Examination, *N* sample size, *NS* not significant, *pos* positive, *SCD* subjective cognitive decline, *SD* standardized deviation

### Cross-sectional data

At baseline, the self-reported SCD differed significantly between the groups for the global_R_ score and the three factors. HC showed lower self-reported SCD than both SCD groups; note that self-reported global_R_ SCD was used to separate HC from SCD-community. As for the informant-reported SCD, SCD-clinic had higher scores than SCD-community and HC, and SCD-community had higher scores than HC, for all SCD measures except for the praxis-domestic activities SCD for which no difference was found between the SCD groups (see Fig. 2b). The two psychoaffective measures showed significant between-group differences: for anxiety, scores were significantly higher in SCD-community and SCD-clinic compared to HC; for depression, SCD-clinic had higher scores than SCD-community and HC (see Fig. 2c, d).

### Substrates of self-reported SCD

#### Cognitive and behavioural correlates

Significant relationships were found between self-reported global_R_ SCD score and each self-reported SCD factors in both SCD groups. Significant correlations were found between self-reported global_R_ SCD and the corresponding measures of informant-reported SCD only in the SCD-clinic. A significant relationship was also found between the self-reported global_R_ SCD score and anxiety in the SCD-community group, while no relationship was found with baseline objective measures of cognition or depression in any group (Table 2).

**Table 2:** Results of linear regressions or general linear models between self-reported global_R_ SCD and cross-sectional measures

	SCD-community		SCD-clinic	
	*r*	*p*	*r*	*p*
Self-reported SCD				
Attention-language SCD (F1)	*0.62*	*0.003*	*0.88*	*< 0.001*
Memory-orientation SCD (F2)	*0.70*	*< 0.001*	*0.90*	*< 0.001*
Praxis-domestic activities SCD (F3)	*0.56*	*0.008*	*0.67*	*< 0.001*
Informant-reported SCD				
Global_R_ SCD	0.10	0.66	*0.68*	*< 0.001*
Cognitive measures				
DRS	− 0.10	0.67	0.14	0.48
ESR	− 0.25	0.25	− 0.14	0.48
Psychoaffective measures				
STAI-B	*0.49*	*0.02*	− 0.03	0.90
MADRS	− 0.01	0.96	− 0.15	0.47

For the informant-reported SCD and cognitive measures, values indicate the results of the simple linear regressions (*r* and *p* values) between the self-reported global_R_ SCD score on the one hand and the corresponding variables on the other hand. For the psychoaffective measures, values indicate the results of the general linear model between the self-reported global_R_ SCD score and these measures, correcting for age and education. Values indicated in italics correspond to *p* < 0.05

*Abbreviations: DRS w*-score of Mattis Dementia Rating Scale, *ESR w*-score of Encoding, Storage and Recuperation, *F1 w*-score of cognitive difficulties scale factor 1 attention-language SCD, *F2 w*-score of cognitive difficulties scale factor 2 memory-orientation SCD, *F3 w*-score of cognitive difficulties scale factor 3 praxis-domestic activities SCD, *MADRS* Montgomery-Asberg Depression Rating Scale, *SCD* subjective cognitive decline, *STAI-B* Spielberger State-Anxiety Inventory Trait

#### Brain correlates

**Fig. 3:**
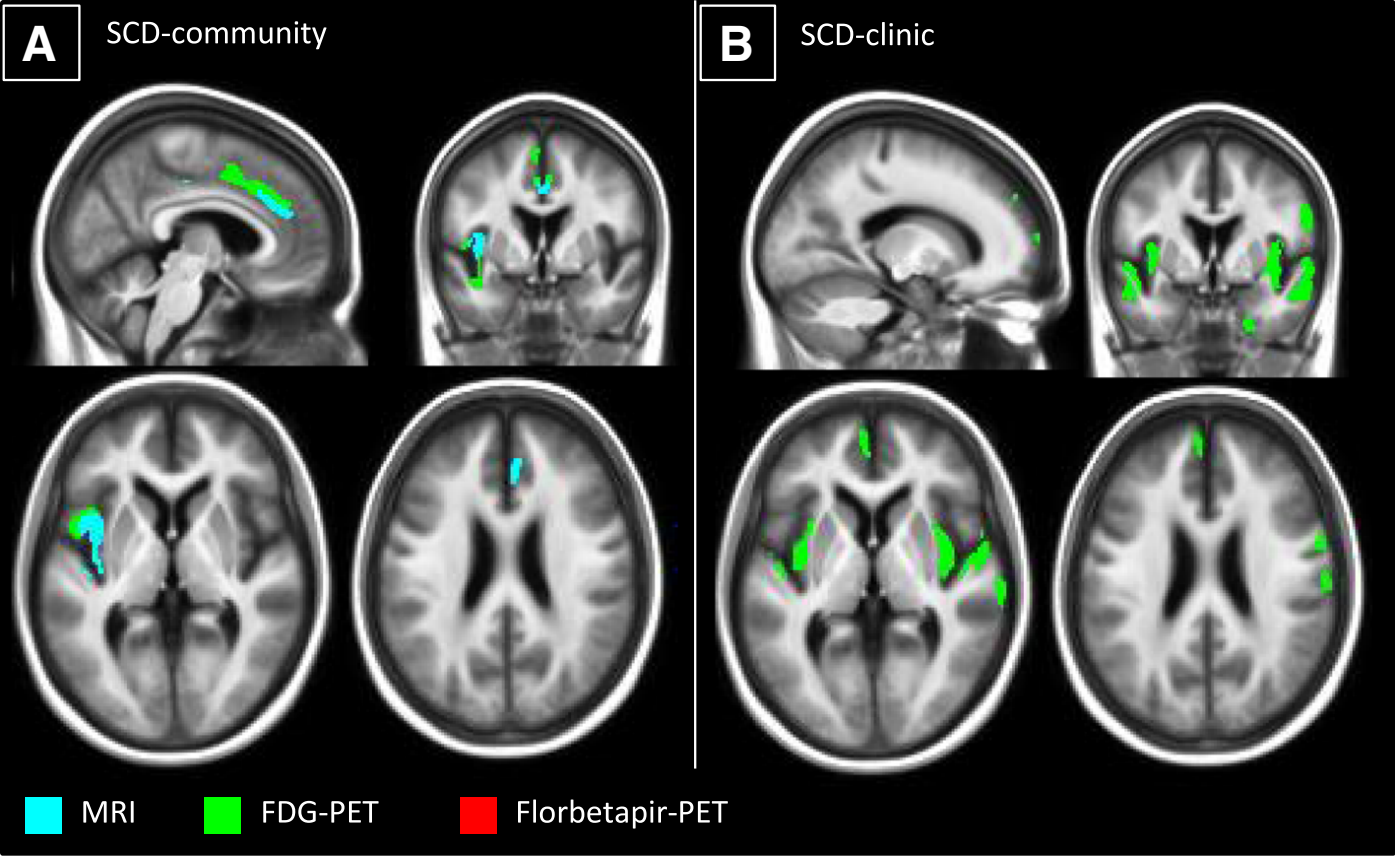
Results of the voxelwise correlations between self-reported global_R_ SCD and neuroimaging within each SCD group. The correlations with grey matter volume (blue), glucose metabolism (green), and amyloid deposition (red) are presented within the SCD-community (**a**) and the SCD-clinic (**b**) groups. The results are displayed at uncorrected *p* < 0.005, *k* > 250 voxels. FDG, 18F-fluorodeoxyglucose; PET, positron emission tomography; SCD, subjective cognitive decline

In the SCD-community group, self-reported global_R_ SCD negatively correlated with glucose metabolism and grey matter volume in the left insula, right superior frontal, and anterior cingulate cortex (Fig. 3a). Except for the right superior frontal correlation with grey matter volume, all clusters were recovered at *p* < 0.001, *k* > 50 voxels (Additional file 3).

In the SCD-clinic group, the self-reported global_R_ SCD negatively correlated with glucose metabolism in the bilateral insula, left medial prefrontal cortex (encompassing both the ventral and dorsal sections), bilateral superior and middle temporal cortex, and right fusiform gyrus. All clusters were recovered at *p* < 0.001, *k* > 50 voxels, except for the left medial prefrontal cortex (Additional file 3). At the threshold of *p* < 0.005 and *k* > 250 voxels, no correlation was found with amyloid deposition or grey matter volume (see Fig. 3b).

For the sake of comparison, the correlations were also assessed with the SCD factors; they showed that the brain substrates of the self-reported memory SCD score were very similar to those of the global_R_ SCD score (Additional file 3).

### Longitudinal data

#### Cognitive and behavioural measures

The self-reported praxis-domestic activities SCD slope was significantly higher than zero (*p* = 0.04), i.e. this measure significantly increased over time, only in the SCD-clinic. By contrast, the depression score slope tended to be higher than zero (*p* = 0.06) only in the SCD-community. None of the other slopes significantly differed from zero indicating that none of the other self- and informant-reported SCD factors and cognitive or psychoaffective measures significantly changed over time during the follow-up period of the SCD groups (Table 3).

**Table 3:** Description of the progression over time of SCD, cognitive, and psychoaffective measures within each group

Scores	*N*	SCD-community	*N*	SCD-clinic	ANCOVAs, *p* value
Follow-up duration, years (behavioural measures)	22	2.45 ± 0.67	25	2.36 ± 0.95	0.32
Follow-up duration, years (neuroimaging measures—MRI)	22	2.55 ± 0.67	24	2.28 ± 0.78	0.22
Self-reported SCD slopes					
Global_R_	22	− 0.08 ± 0.27	20	0.08 ± 0.35	0.11
Attention-language (F1)	22	0.009 ± 0.06	20	0.02 ± 0.11	0.60
Memory-orientation (F2)	22	− 0.03 ± 0.09	20	0.004 ± 0.12	0.28
Praxis-domestic activities (F3)	22	− 0.009 ± 0.07	20	0.04 ± 0.09^#^	*0.04*
Informant-reported SCD slopes					
Global_R_	15	0.06 ± 0.46	16	0.07 ± 0.65	0.98
Attention-language (F1)	15	− 0.02 ± 0.13	16	0.01 ± 0.2	0.65
Memory-orientation (F2)	15	0.02 ± 0.12	16	− 0.007 ± 0.12	0.61
Praxis-domestic activities (F3)	15	0.03 ± 0.15	16	0.02 ± 0.1	0.94
Slope of cognitive change					
DRS	22	− 0.02 ± 0.14	25	− 0.03 ± 0.11	0.74
ESR	22	0.0005 ± 0.03	24	− 0.02 ± 0.09	0.27
Slope of psychoaffective changes					
STAI-B	22	− 0.04 ± 0.21	25	0.05 ± 0.22	0.16
MADRS	22	0.09 ± 0.20	23	0.08 ± 0.26	0.92

The values indicate the mean ± SD of the slope of evolution between baseline and follow-up. When ANCOVAs correcting for age and education reached significance, values are indicated in italics (*p* < 0.05)

*Abbreviations*: *ANCOVAs* analyses of covariance correcting for age and education, *CDS* Cognitive Difficulties Scale, *DRS* Mattis Dementia Rating Scale, *ESR* Encoding, Storage and Recuperation, *F1* factor 1 attention-language SCD, *F2* factor 2 memory-orientation SCD, *F3* factor 3 praxis-domestic activities SCD, *HC* healthy control, *MADRS* Montgomery-Asberg Depression Rating Scale, *MRI* magnetic resonance imaging, *N* sample size, *SCD* subjective cognitive decline, *SD* standardized deviation, *STAI-B* Spielberger State-Anxiety Inventory Trait

^#^The slope is significantly different from zero (*p* < 0.05)

At follow-up, there was no significant group difference for any cognitive, psychoaffective, or SCD slope of changes, except for the self-reported praxis-domestic activities SCD which increased more in the SCD-clinic group than in the SCD-community group (Table 3).

#### Atrophy progression over time in MRI

**Fig. 4:**
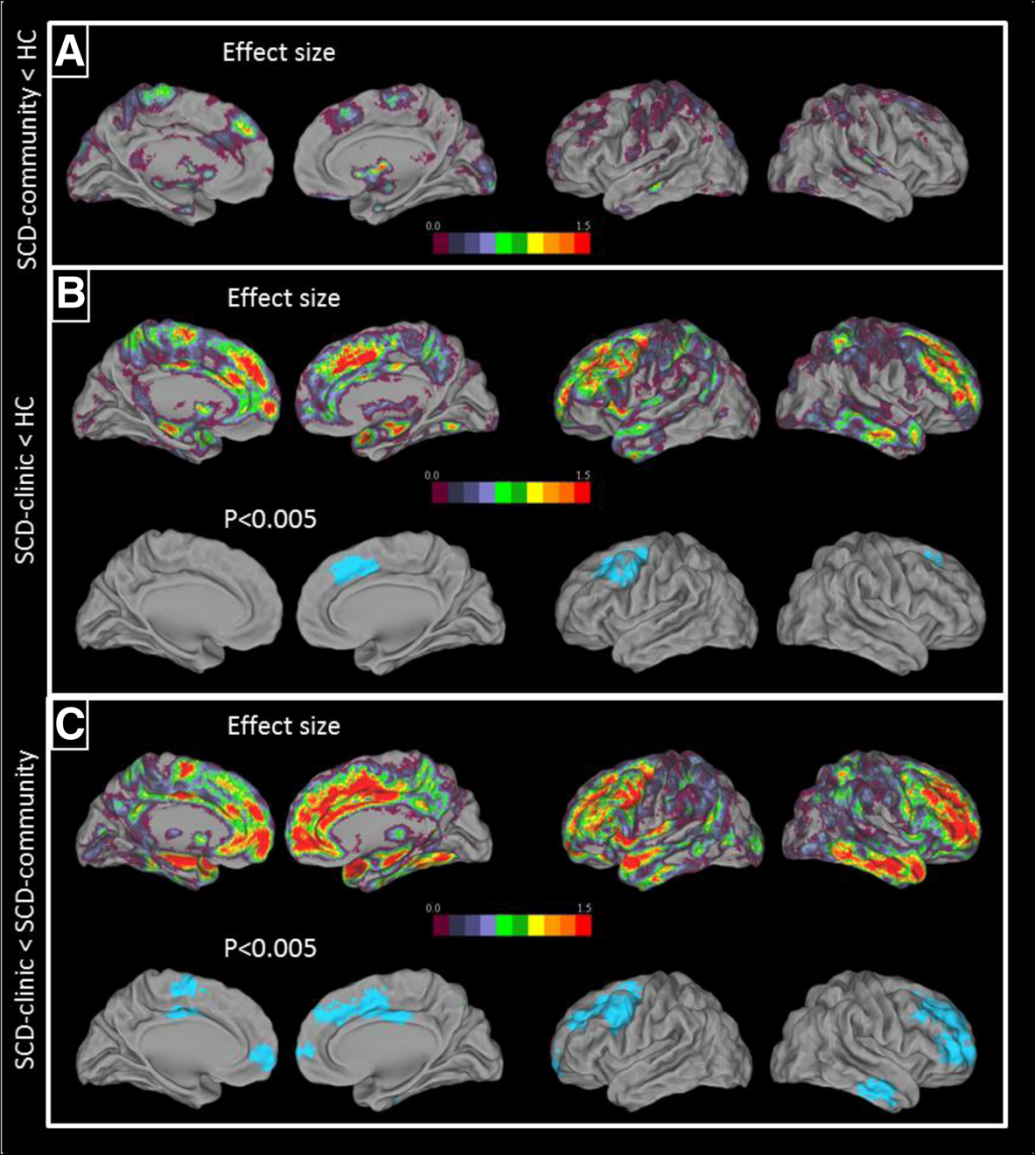
Results of the between-group comparisons of brain maps of atrophy progression over time. Voxelwise comparisons showed the regions of significantly higher atrophy progression over time in SCD-community as compared to healthy control (HC) (**a**) and in SCD-clinic as compared to HC (**b**) and to SCD-community (**c**). The results are displayed as *T* value maps thresholded at uncorrected *p* < 0.005, *k* > 250 voxels and as effect size maps. SCD, subjective cognitive decline

Between-group comparisons of brain maps of atrophy progression over time showed that SCD-clinic had higher atrophy progression over time in the dorsal frontal cortex compared to HC (Fig. 4b) and in the middle temporal cortex and dorsal frontal cortex extending to the ventral prefrontal cortex compared to SCD-community (Fig. 4c). There was no significant difference in the atrophy progression over time between HC and SCD-community (Fig. 4a). All clusters were recovered at *p* < 0.001, *k* > 50 voxels (Additional file 4).

#### Predictors of cognitive decline

Regression analyses between the slope of cognitive decline and baseline measures showed that a high self-reported global_R_ (*r* = − 0.59, *p* = 0.007) or memory (*r* = − 0.52, *p* = 0.02) SCD at baseline correlated with a higher global cognitive decline only in the SCD-community group. No baseline measures predicted the evolution of cognitive performances in the SCD-clinic group (Additional file 5).

## Discussion

The type of recruitment of SCD patients might have a significant impact on the characteristics, aetiology, and risk of Alzheimer’s clinical syndrome of the recruited SCD sample. Yet, the characteristics specifically associated with help-seeking behaviour in SCD have only been assessed in a few studies [25, 72–76], and most of these studies were not restricted to elders with formally assessed normal cognitive performances, few of them included the role of the informant or neuroimaging biomarkers and none included longitudinal data. This study thus aimed at identifying the characteristics of SCD individuals according to their type of recruitment (SCD-community versus SCD-clinic) in terms of cross-sectional and longitudinal self- and informant-reported SCD, subclinical anxiety and depression, cognitive performances, and atrophy progression over time, all known to increase the risk of being at a preclinical stage of Alzheimer’s syndrome [1, 43, 49–54, 77, 78]. We also assessed the substrates of self-reported SCD within each group to identify the main respective drivers of their SCD which could include risk factor of Alzheimer’s clinical syndrome [25] or psychological distress [39].

### Common points between the SCD groups

The two SCD groups presented similar characteristics, although the recruitment setting differs. Thus, at a comparable level of self-reported global_R_ SCD, both SCD groups showed a higher level of subclinical anxiety and a higher level of informant-reported global_R_ SCD but similar cognitive performances compared to controls. Hence, in both SCD groups, the self-reported global_R_ SCD correlated to the three factors—suggesting that they are not driven by the subjective decline in the distinct cognitive domain—but surprisingly not to any objective measure of cognition. This highlights the relevance of the self- and informant-reported SCD, which might be sensitive to subtle cognitive changes not yet detectable using objective measures [46, 49, 50]. No significant change in cognition was found in any group, which might reflect the fact that the follow-up period (2.4 ± 0.8 years) was too short to capture subtle cognitive decline.

Surprisingly, our findings also showed no difference between the groups in term of amyloid status or SUVr uptake values. In contrast to previous studies, the self-reported SCD was not associated with the presence of amyloid deposition in cognitively unimpaired elders recruited from the community [18, 20, 21, 23, 25] or in SCD-clinic patients [22, 25]. However, this effect seems subtle and might depend on the sample and the measurement used [21, 22], as other studies have reported negative results like ours [79, 80] compared with controls, or found an association only in APOE ε4 carriers [81, 82]. Interestingly, when merging our two SCD groups together, a significant between-group difference was found with higher amyloid SUVr in the SCD compared to the controls (*p* = 0.044, Additional file 6). The lack of difference between the SCD groups might also be due to the fact that the SCD-clinic tended to be younger (about 68.3 years old in SCD-clinic against 71.70 in SCD-community and 72.25 in controls), and the proportion of APOE ε4 carriers was relatively small in the SCD-clinic (15%) compared to the SCD-community (26%) or the controls (18%); when the analyses were corrected for age, level of education, and APOE ε4 status, a general trend appeared in the between-group comparison of amyloid SUVr (*p* = 0.096, Additional file 6).

### Specificities of both SCD groups

#### Specificities of the SCD-community

While the SCD-community share similarities with the SCD-clinic, our findings also highlight the differences. Thus, the SCD-community showed a higher subclinical anxiety than older adults without subjective cognitive decline. Moreover, their anxiety score correlated with their level of self-reported global_R_ SCD which itself predicted their subsequent cognitive decline. This suggests that anxiety contributes to the level of SCD only in the SCD-community group, while psychoaffective factors do not influence SCD in the SCD-clinic group. Neuroimaging correlates confirmed this view in showing a link between SCD and frontal grey matter volume and glucose metabolism in SCD-community, while it rather involved the temporal and parietal brain regions sensitive to AD [19, 57, 83] in the SCD-clinic group. As suggested in previous studies [39, 84–86], the subjective cognitive decline of SCD-community thus seems to be more strongly related to the psychoaffective factors.

#### SCD-clinic seems to be a frailer population than SCD-community

By contrast, a few evidence suggest that SCD-clinic might be a frailer population than SCD-community. Thus, cognitively unimpaired older adults with subjective cognitive decline who referred to a memory clinic were characterized by higher informant-reported global_R_ SCD, higher depressive symptoms, and greater subsequent atrophy progression over time than those with the same level of SCD but who did not refer to a memory clinic. Moreover, SCD-clinic tended to show a more generalized profile of SCD. Indeed, while their level of self-reported SCD was similar to SCD-community, they showed a significant increase in praxis-domestic activities SCD over the follow-up period.

In a previous study [25], we showed that subclinical depression and (notably hippocampal) atrophy were specifically associated with medical help-seeking, suggesting that those who consult are at a higher risk of developing Alzheimer’s clinical syndrome, as indicated in another study which compared the rates of incident dementia [37]. In the present study, our findings reinforced this view by showing greater atrophy progression over time. The atrophy progression over time was significantly higher in the frontal cortex and tended to be higher in the temporal lobe and particularly in the hippocampus (see effect size on Fig. 4). It might thus reflect accelerated brain ageing related to psychoaffective factors, as the frontal areas are known to be sensitive to ageing [84–86] and commonly associated with subclinical anxiety [87] and depression [88]—themselves associated with increased risk for cognitive decline or dementia [27, 33]. These findings might also reflect, to a lesser extent, increased risk for dementia, as greater hippocampal atrophy progression over time is known to be associated with subsequent cognitive decline [89] and dementia [90]. Similarly, the fact that they showed greater praxis-domestic activities SCD over time might also represent an additional evidence for this statement. Thus, as multi-domain amnestic MCI are known to be more at risk of AD than single-domain MCI [91], the generalization of SCD in the SCD-clinic group might alike indicate that they are in a more advanced stage of SCD and possibly represent a frailer population than SCD-community, with an increased risk of cognitive decline, and potentially Alzheimer’s clinical syndrome. These are yet only indirect evidences and longer-term follow-up in larger samples are needed to confirm this hypothesis.

### SCD-community and SCD-clinic: a continuum or distinct entities?

One possible interpretation of our findings is that SCD-community represents an intermediate stage in a continuum leading to SCD-clinic. Indeed, they showed intermediate levels of informant-reported global_R_ SCD, and their level of subclinical depression tended to increase at follow-up (*p* = 0.06), reaching the level of depression of the SCD-clinic (*p* = 0.25, data not shown). While their neural correlates for self-reported global_R_ SCD were different from SCD-clinic for some brain regions, there were also common functional brain correlates (in the insula and frontal cortex) between both SCD groups.

The intermediate level of informant-reported global_R_ SCD in the SCD-community highlights the sensitivity of this measure to capture subtle differences between the SCD groups that refer or not to a memory clinic. Previous studies have shown that the informant-reported SCD can be associated with longitudinal cognitive decline [49] and a higher risk of subsequent conversion to MCI or AD dementia [50, 92]; suggesting that both groups show risk factors for Alzheimer’s clinical syndrome. Moreover, this is consistent with the fact that the self-reported SCD measure predicted the level of subsequent cognitive decline in the SCD-community, adding to the risk.

As regards to psychoaffective factors, a higher level of subclinical anxiety compared to controls characterized both SCD groups, while the level of subclinical depression was higher only in the SCD-clinic group at baseline and increased from baseline to follow-up in the SCD-community. As a whole, our findings suggest that referring to a memory clinic is associated with subclinical depression rather than with the level of subclinical anxiety or self-reported SCD. Interestingly, both psychoaffective factors (depressive symptoms and subclinical anxiety) are frequently associated with early cognitive deficits [53, 93, 94] or subsequent dementia [51, 54] and could be a prodromal sign of Alzheimer’s clinical syndrome [55]. However, the causal link between self- and informant-reported SCD, psychoaffective factors, and cognitive or brain changes is unclear. SCD and frontal atrophy might lead to an increase in subclinical anxiety and depression, themselves associated with an increase in informant-reported SCD, leading to memory consultation. Alternatively, psychoaffective factors might lead to, or exacerbate, brain and cognitive decline underlying self- and informant-reported SCD and stimulating memory consultation. Further studies are needed to better understand the sequence of events and causal relationships between these different factors. Specifically, a better understanding of the role of psychoaffective factors is important for the development of non-pharmacological interventions targeting emotional regulation processes [95].

Altogether, our results suggest that the two SCD groups have specificities but may represent in fact different stages of progressive cognitive decline that may lead to Alzheimer’s clinical syndrome. As for early and late MCI [28, 96], SCD-community and SCD-clinic might represent two stages of SCD in a continuum that would lead, for part of them, to Alzheimer’s clinical syndrome. However, we cannot exclude the alternative hypothesis that, instead of a continuum, the two groups represent distinct selections of individuals with SCD where underlying neuropsychiatric/non-AD aetiologies versus AD pathology are differently represented.

### Strengths, limitations, and perspectives

The major strengths of this study are its multimodal dimension and the combination of cross-sectional and longitudinal setup. Indeed, the availability of standardized assessment of a broad range of factors potentially related to SCD, including various biomarkers, provides novel insights into the integrated characterization of SCD in the context of preclinical AD/Alzheimer’s syndrome. However, although based on the recommendations of Jessen et al., 2014, which list the specific features that increase the likelihood of the presence of preclinical AD/Alzheimer’s syndrome in individuals with SCD [1, 45] and a previous study [56], the threshold used to separate SCD-community from controls was somewhat arbitrary. In addition, all the questionnaires were self-completed, and the sample sizes and follow-up time were relatively limited, resulting in a limited statistical power. Consequently, the statistics were not corrected for multiple comparisons which increase the risk for false positive. Therefore, our results should be interpreted with caution and validated in future studies with larger group sizes and longer follow-up time. This would also allow the assessment of the sequential and causal relationships between the different factors to understand the role of psychoaffective factors and informant-reported SCD, but also to confirm that SCD-community and SCD-clinic are two stages of a continuum in preclinical Alzheimer’s syndrome. Nowadays, characterizing and discriminating preclinical Alzheimer’s syndrome from the ‘worried well’ seems especially important for the early detection of persons in the preclinical stage of dementia, the prevention, and the development of efficient therapies. Given the heterogeneity of the aetiology and presentation of SCD [1, 25, 45, 46], a better understanding of these two populations might help us to identify the potential targets for pharmacological or non-pharmacological interventions.

## Conclusions

As a whole, our results point to the SCD-clinic as a frailer population showing faster atrophy over time, compared to HC and SCD-community, which might reflect an increased risk for later cognitive decline. Depression symptoms were also higher in the SCD-clinic, but they increased over time in the SCD-community, suggesting a continuity between SCD groups. Alternatively, they might reflect distinct populations with different proportion of possible aetiologies (AD pathology, neuropsychiatric aetiologies, etc.). From a clinical standpoint, SCD patients might thus benefit from a closer clinical follow-up; from a research standpoint, this population could enrich interventional clinical trials on SCD with more participants at risk of AD/dementia. Finally, our findings highlight the relevance of psychoaffective factors, including both subclinical anxiety and depression, at this stage. Rather than confounding factors of SCD, psychoaffective factors might represent early symptoms of Alzheimer’s clinical syndrome, or even the expression of a pathological process associated with psychological distress and related to subsequent cognitive decline in the SCD-community. This, along with the fact that they are associated with increased risk of dementia, highlights the relevance of treating these symptoms in the elderly. Further studies are yet needed to better understand their causal and/or consequent role within the different SCD stages.

## Additional files

Additional file 1: Population sizes at the various imaging examination time points: cross-sectional and longitudinal neuroimaging assessments. Abbreviations: HC healthy control, SCD subjective cognitive decline, *N* sample size, MRI magnetic resonance imaging, PET positron emission tomography, FDG 18F-fluorodeoxyglucose. (DOCX 46 kb)

Additional file 2: Description of the methodology of the longitudinal neuroimaging data processing. (DOCX 16 kb)

Additional file 3: Glass brain of the voxelwise correlations between self-reported SCD and neuroimaging within each SCD group. The correlations between self-reported SCD measures (1, Global_R_ SCD; 2, Attention/Language SCD; 3, Memory/Orientation SCD; 4, Praxis/Domestic Activities SCD) and grey matter volume (MRI), glucose metabolism (FDG-PET), or amyloid deposition (Florbetapir-PET) are presented within the SCD-community (A) and the SCD-clinic (B) groups. The results are displayed at uncorrected *p* < 0.005, *k* > 250 voxels and *p* < 0.001, *k* > 50 voxels for all analyses. FDG 18F-fluorodeoxyglucose, PET positron emission tomography, SCD subjective cognitive decline. (DOCX 538 kb)

Additional file 4: Glass brains of the between-group comparisons of brain maps of atrophy progression over time. Voxelwise comparisons show the regions of significantly higher atrophy progression over time in SCD-community as compared to healthy control (HC) (A), and in SCD-clinic as compared to HC (B) and to SCD-community (C). The results are displayed as *T* value maps thresholded at uncorrected *p* < 0.005, *k* > 250 voxels and *p* < 0.001, *k* > 50 voxels. SCD Subjective cognitive decline. (DOCX 267 kb)

Additional file 5: Results of the general linear model assessing the links between baseline variables and cognitive decline slopes. The values indicate the results of the general linear model assessing the links between baseline self- and informant-reported SCD factors or psychoaffective measures and the slope of cognitive decline, corrected for age and education. Values indicated in bold correspond to *p* < 0.05. Abbreviations: DRS Mattis Dementia Rating Scale, ESR Encoding, Storage and Recuperation, *F1* score of cognitive difficulties scale factor 1 attention-language SCD, *F2* score of cognitive difficulties scale factor 2 memory-orientation SCD, *F3* score of cognitive difficulties scale factor 3 praxis-domestic activities SCD, MADRS Montgomery-Asberg Depression Rating Scale, SCD subjective cognitive decline, STAI-B Spielberger State-Anxiety Inventory Trait. (DOCX 21 kb)

Additional file 6: Group comparisons on amyloid SUVr. Graphs indicate mean values and 95% confidence intervals. A: three-group comparisons on the amyloid SUVr and post-hoc analyses performed with the Newman-Keuls test; B: two-group comparisons on the amyloid SUVr, when SCD groups were merged. ANCOVA analysis of variance corrected, APOE apolipoprotein E, HC healthy control, SCD subjective cognitive decline, SUVr standardized uptake value ratio. (DOCX 77 kb)

## Data Availability

The datasets generated and analysed during the current study are not publicly available due to local privacy regulations but are available from the corresponding author on reasonable request.

## Abbreviations

AD: Alzheimer’s disease
ANCOVA: Analysis of covariance
ANOVA: Analysis of variance
APOE ε4: Apolipoprotein E allele 4
Aβ: Amyloid deposition
CDS: Cognitive Difficulties Scale
DRS: Mattis Dementia Rating Scale
ESR: Encoding, Storage and Recuperation
F1: Factor 1 of CDS corresponding to attention-language SCD
F2: Factor 2 of CDS corresponding to memory-orientation SCD
F3: Factor 3 of CDS corresponding to praxis-domestic activities SCD
FDG: 18F-fluorodeoxyglucose
FWHM: Full-width half-maximum
HC: Healthy controls
IMAP+: *Imagerie Multimodale de la maladie d’Alzheimer Précoce*
MADRS: Montgomery-Asberg Depression Rating Scale
MCI: Mild cognitive impairment
MMSE: Mini-Mental State Examination
MNI: Montreal Neurological Institute
MRI: Magnetic resonance imaging
*N*: Size
NS: Not significant
PET: Positron emission tomography
Pos: Positive
SCD: Subjective cognitive decline
SCD-clinic: Subjective cognitive decline recruited from a memory clinic
SCD-community: Subjective cognitive decline recruited from volunteers from the community
SCD-I: Subjective Cognitive Decline Initiative
SD: Standard deviation
SPM12: Statistical Parametric Mapping version 12
STAI-B: Spielberger State-Trait Anxiety Inventory-B
SUVr: Standardized uptake value ratio
