# Supplementary material for: Cross-sectional and longitudinal characterization of SCD patients recruited from the community versus from a memory clinic: subjective cognitive decline, psychoaffective factors, cognitive performances, and atrophy progression over time
Source: Alzheimers Res Ther. 2019 Jul 8;11:61. doi: 10.1186/s13195-019-0514-z (PMC6615169; doi:10.1186/s13195-019-0514-z)
Supplement: Supplementary file 1 — Population sizes at the various imaging examination time points: cross-sectional and longitudinal neuroimaging assessments. Abbreviations: HC healthy control, SCD subjective cognitive decline, N sample size, MRI magnetic resonance imaging, PET positron emission tomography, FDG 18F-fluorodeoxyglucose. (DOCX 46 kb) [file 13195_2019_514_MOESM1_ESM.docx]

| **N** | **HC** | **SCD-community** | **SCD-clinic** |
| --- | --- | --- | --- |
| Cross-sectional neuroimaging assessment | | | |
| MRI | 28 | 23 | 27 |
| FDG-PET | 27 | 23 | 27 |
| Florbetapir-PET | 25 | 22 | 27 |
| Longitudinal neuroimaging assessment | | | |
| Maps of atrophy progression over time | 27 | 22 | 24 |
