# Supplementary material for: Cross-sectional and longitudinal characterization of SCD patients recruited from the community versus from a memory clinic: subjective cognitive decline, psychoaffective factors, cognitive performances, and atrophy progression over time
Source: Alzheimers Res Ther. 2019 Jul 8;11:61. doi: 10.1186/s13195-019-0514-z (PMC6615169; doi:10.1186/s13195-019-0514-z)
Supplement: Supplementary file 2 — Description of the methodology of the longitudinal neuroimaging data processing. (DOCX 16 kb) [file 13195_2019_514_MOESM2_ESM.docx]

For each participant, a pairwise longitudinal registration was computed between the baseline and follow-up anatomical MRI resulting in a mid-point average anatomical MRI image (anatomical MRI average) and the Jacobians. The Jacobian rates were computed by differences between the Jacobian determinants, divided by the time interval between the two scans, and reflect a rate of change between baseline and follow-up MRI scans. Values of Jacobian rates less than zero indicate contraction (over time), whereas values greater than zero indicate expansion [1]. The anatomical MRI average was segmented into grey matter, white matter and cerebrospinal fluid. The resulting grey matter segment (reflecting an average grey matter density probability map) was then (i) multiplied by the Jacobian rates resulting in the grey matter change map (reflecting a map of grey matter probability changes between baseline and follow-up); and (ii) normalized to the DARTEL template (Diffeomorphic Anatomical Registration through Exponentiated Lie Algebra; the Template was obtained from healthy volunteers and patients from IMAP+); the resulting deformation field was then applied to the grey matter change map. The normalized grey matter change map, smoothed with an 8mm full-width at half-maximum isotropic Gaussian kernel, was used as a reflect of atrophy propagation progression (i.e. atrophy rate maps) over the follow-up period for each subject [2].

**References**

1. Ashburner J, Barnes G, Chen C, Daunizeau J, Moran R, Henson R, et al. SPM12 Manual The FIL Methods Group (and honorary members). London: Functional Imaging Laboratory. 2016.

2. Mutlu J, Landeau B, Gaubert M, de La Sayette V, Desgranges B, Chételat G. Distinct influence of specific versus global connectivity on the different Alzheimer’s disease biomarkers. Brain J Neurol. 2017;140:3317–28.
