# Supplementary figures and images for: Cross-sectional and longitudinal characterization of SCD patients recruited from the community versus from a memory clinic: subjective cognitive decline, psychoaffective factors, cognitive performances, and atrophy progression over time
Source: Alzheimers Res Ther. 2019 Jul 8;11:61. doi: 10.1186/s13195-019-0514-z (PMC6615169; doi:10.1186/s13195-019-0514-z)

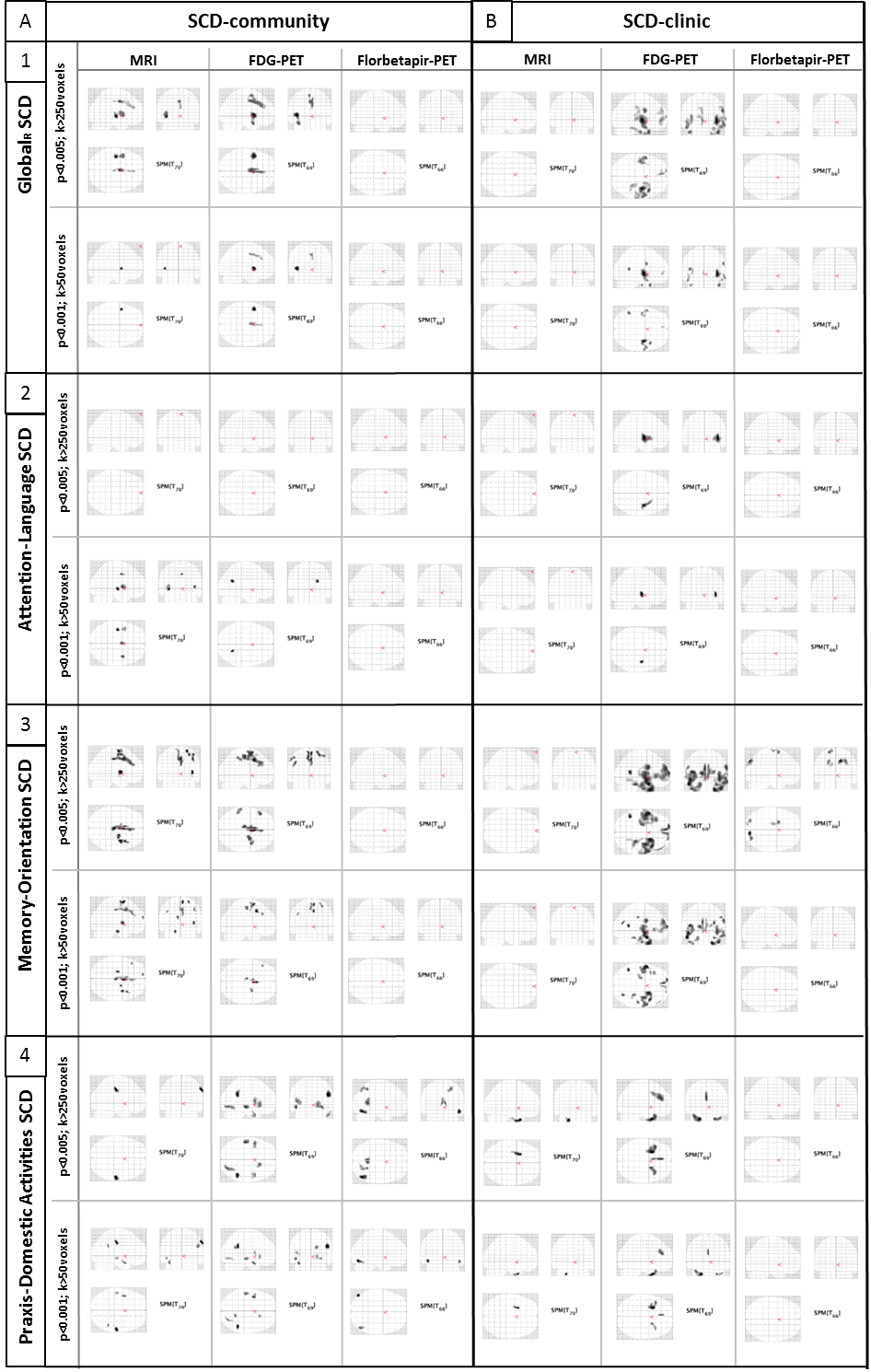

Supplement: Supplementary file 3 — Glass brain of the voxelwise correlations between self-reported SCD and neuroimaging within each SCD group. The correlations between self-reported SCD measures (1, GlobalR SCD; 2, Attention/Language SCD; 3, Memory/Orientation SCD; 4, Praxis/Domestic Activities SCD) and grey matter volume (MRI), glucose metabolism (FDG-PET), or amyloid deposition (Florbetapir-PET) are presented within the SCD-community (A) and the SCD-clinic (B) groups. The results are displayed at uncorrected p < 0.005, k > 250 voxels and p < 0.001, k > 50 voxels for all analyses. FDG 18F-fluorodeoxyglucose, PET positron emission tomography, SCD subjective cognitive decline. (DOCX 538 kb) [file 13195_2019_514_MOESM3_ESM.docx]

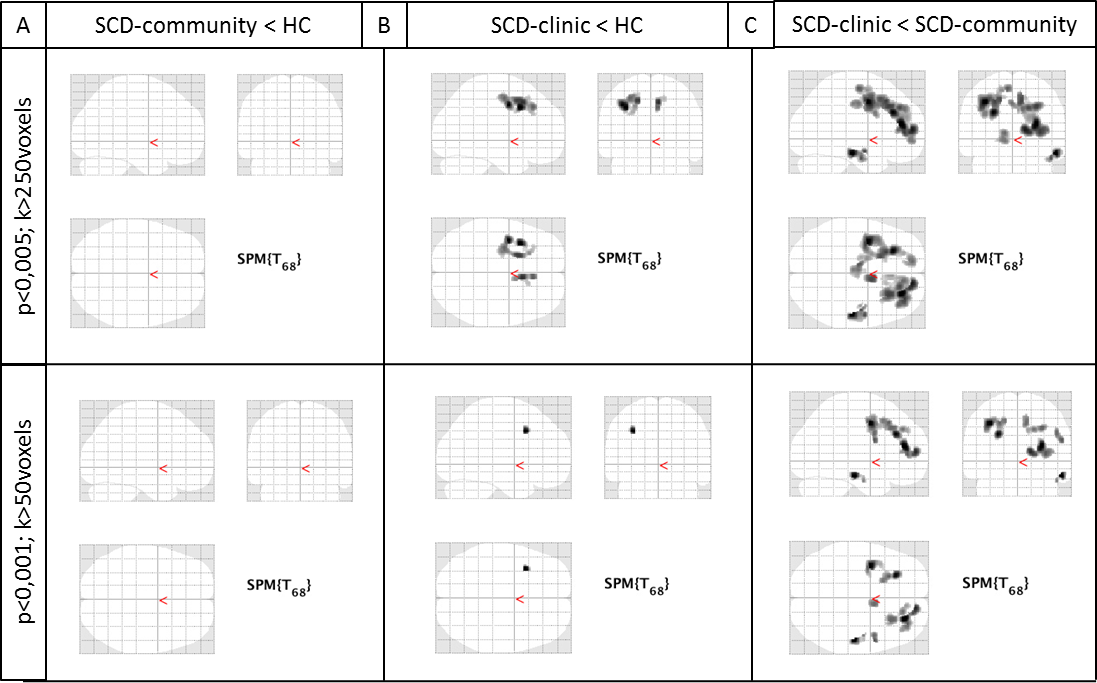

Supplement: Supplementary file 4 — Glass brains of the between-group comparisons of brain maps of atrophy progression over time. Voxelwise comparisons show the regions of significantly higher atrophy progression over time in SCD-community as compared to healthy control (HC) (A), and in SCD-clinic as compared to HC (B) and to SCD-community (C). The results are displayed as T value maps thresholded at uncorrected p < 0.005, k > 250 voxels and p < 0.001, k > 50 voxels. SCD Subjective cognitive decline. (DOCX 267 kb) [file 13195_2019_514_MOESM4_ESM.docx]

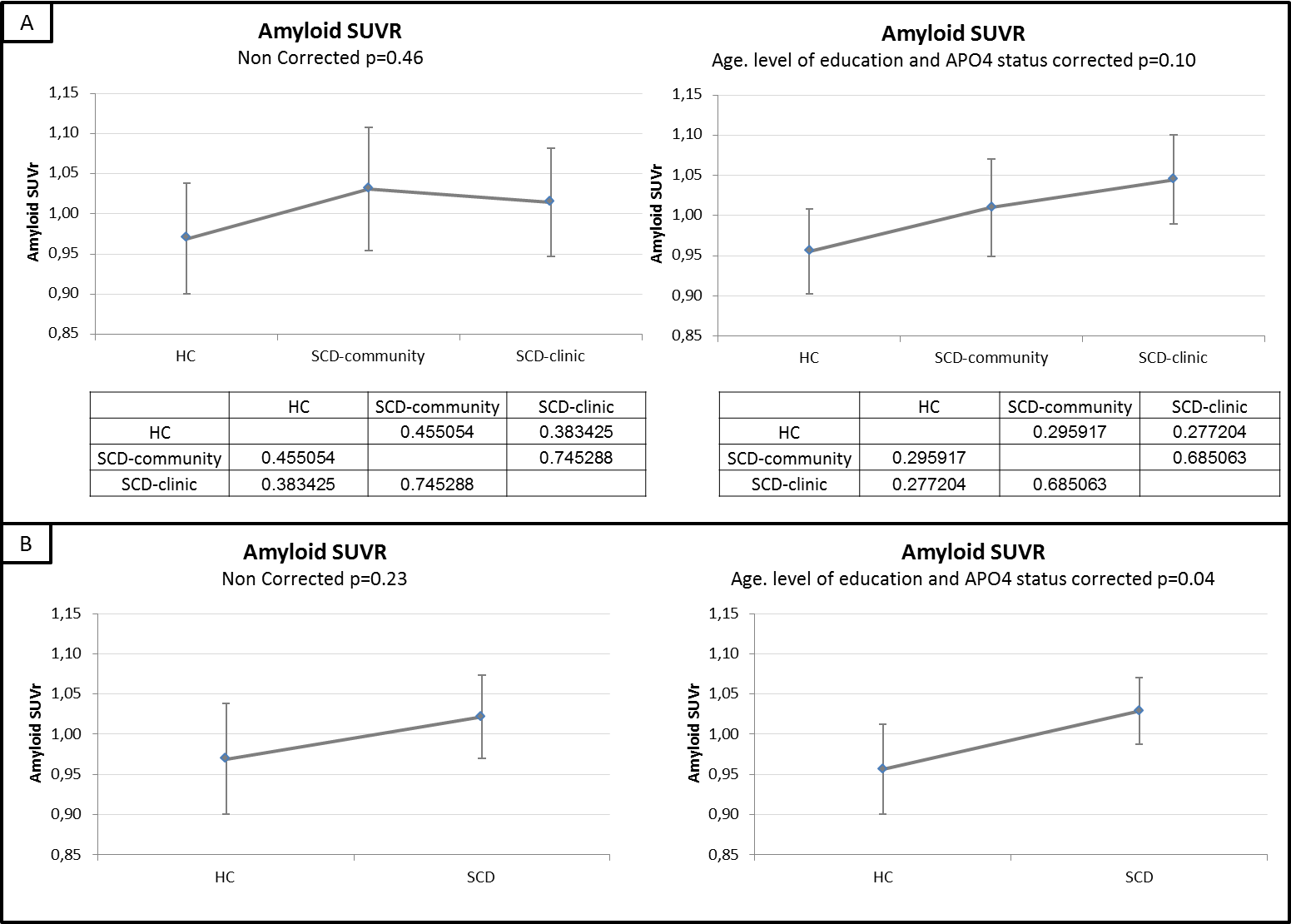

Supplement: Supplementary file 6 — Group comparisons on amyloid SUVr. Graphs indicate mean values and 95% confidence intervals. A: three-group comparisons on the amyloid SUVr and post-hoc analyses performed with the Newman-Keuls test; B: two-group comparisons on the amyloid SUVr, when SCD groups were merged. ANCOVA analysis of variance corrected, APOE apolipoprotein E, HC healthy control, SCD subjective cognitive decline, SUVr standardized uptake value ratio. (DOCX 77 kb) [file 13195_2019_514_MOESM6_ESM.docx]
