# Supplementary material for: Cross-sectional and longitudinal characterization of SCD patients recruited from the community versus from a memory clinic: subjective cognitive decline, psychoaffective factors, cognitive performances, and atrophy progression over time
Source: Alzheimers Res Ther. 2019 Jul 8;11:61. doi: 10.1186/s13195-019-0514-z (PMC6615169; doi:10.1186/s13195-019-0514-z)
Supplement: Supplementary file 5 — Results of the general linear model assessing the links between baseline variables and cognitive decline slopes. The values indicate the results of the general linear model assessing the links between baseline self- and informant-reported SCD factors or psychoaffective measures and the slope of cognitive decline, corrected for age and education. Values indicated in bold correspond to p < 0.05. Abbreviations: DRS Mattis Dementia Rating Scale, ESR Encoding, Storage and Recuperation, F1 score of cognitive difficulties scale factor 1 attention-language SCD, F2 score of cognitive difficulties scale factor 2 memory-orientation SCD, F3 score of cognitive difficulties scale factor 3 praxis-domestic activities SCD, MADRS Montgomery-Asberg Depression Rating Scale, SCD subjective cognitive decline, STAI-B Spielberger State-Anxiety Inventory Trait. (DOCX 21 kb) [file 13195_2019_514_MOESM5_ESM.docx]

|  | Slope of decline DRS | | Slope of decline ESR | |
| --- | --- | --- | --- | --- |
|  | r | p | r | p |
| **SCD-community** |  |  |  |  |
| Baseline self-reported SCD | | | | |
| Global_R_ SCD | **-0,59** | **0.007** | -0.13 | 0.57 |
| Attention-language SCD (F1) | -0.37 | 0.11 | -0.34 | 0.15 |
| Memory-Orientation SCD (F2) | **-0.52** | **0.02** | 0.02 | 0.94 |
| Praxis-Domestic Activities SCD (F3) | -0.22 | 0.35 | 0.18 | 0.46 |
| Baseline informant-reported SCD | | | | |
| Global_R_ SCD | -0.21 | 0.57 | 0.28 | 0.44 |
| Attention-language SCD (F1) | -0.14 | 0.69 | 0.40 | 0.26 |
| Memory-Orientation SCD (F2) | -0.05 | 0.89 | 0.09 | 0.81 |
| Praxis-Domestic Activities SCD (F3) | 0.31 | 0.39 | 0.43 | 0.22 |
| Baseline psychoaffective measures | | | | |
| STAI-B | -0.36 | 0.12 | 0.11 | 0.65 |
| MADRS | 0.15 | 0.52 | 0.20 | 0.40 |
| **SCD-clinic** |  |  |  |  |
| Baseline self-reported SCD | | | | |
| Global_R_ SCD | -0.07 | 0.76 | 0.15 | 0.52 |
| Attention-language SCD (F1) | -0.05 | 0.84 | 0.19 | 0.39 |
| Memory-Orientation SCD (F2) | -0.02 | 0.93 | 0.04 | 0.85 |
| Praxis-Domestic Activities SCD (F3) | -0.08 | 0.70 | 0.14 | 0.53 |
| Baseline informant-reported SCD | | | | |
| Global_R_ SCD | 0.21 | 0.39 | 0.22 | 0.39 |
| Attention-language SCD (F1) | 0.17 | 0.48 | 0.25 | 0.16 |
| Memory-Orientation SCD (F2) | 0.24 | 0.32 | 0.10 | 0.71 |
| Praxis-Domestic Activities SCD (F3) | -0.07 | 0.78 | 0.35 | 0.16 |
| Baseline psychoaffective measures | | | | |
| STAI-B | 0.01 | 0.95 | -0.27 | 0.23 |
| MADRS | 0.08 | 0.73 | -0.15 | 0.52 |
